# Development of a Novel H-Shaped Electrochemical Aptasensor for Detection of Hg^2+^ Based on Graphene Aerogels–Au Nanoparticles Composite

**DOI:** 10.3390/bios13100932

**Published:** 2023-10-18

**Authors:** Gang Peng, Mengxue Guo, Yuting Liu, Han Yang, Zuorui Wen, Xiaojun Chen

**Affiliations:** 1 College of Food Engineering, Anhui Science and Technology University, Fengyang 233100, China; 13696601810@163.com (M.G.); 17855325041@139.com (Y.L.); 17855911091@163.com (H.Y.); wenzr@ahstu.edu.cn (Z.W.); 2 College of Chemistry and Molecular Engineering, Nanjing Tech University, Nanjing 211816, China; chenxj@njtech.edu.cn

**Keywords:** graphene aerogels–Au nanoparticles, Hg^2+^, H-shaped, aptasensor, milk sample

## Abstract

Hg^2+^, a highly toxic heavy metal, poses significant environmental and health risks, necessitating rapid detection methods. In this study, we employed an electrochemical aptasensor for rapid and sensitive detection of Hg^2+^ based on DNA strands (H2 and H3) immobilized graphene aerogels-Au nanoparticles (GAs-AuNPs) hybrid recognition interface and exonuclease III (Exo III)-mediated cyclic amplification. Firstly, Gas-AuNPs were modified on the surface of the ITO electrode to form a sensing interface to increase DNA loading and accelerate electron transfer. Then, DNA helper was generated with the addition of Hg^2+^ via Exo III-mediated cycling. Finally, the hairpin structures of H2 and H3 were opened with the DNA helper, and then the methylene blue (MB) functionalized DNA (A1 and A2) combined with the H2 and H3 to form an H-shaped structure. The current response of MB as an electrochemical probe was proportional to the concentration of Hg^2+^. Under optimal conditions, the aptasensor showed excellent performance for Hg^2+^, achieving a linear range from 1 fM to 10 nM and a detection limit of 0.16 fM. Furthermore, the aptasensor was used to detect Hg^2+^ in spiked milk samples, achieving a high recovery rate and demonstrating promising application prospects.

## 1. Introduction

The issue of heavy metal pollution has emerged as a pressing global environmental concern owing to the continuous progress of industrialization. Heavy metals, including Hg^2+^, Pb^2+^, Cu^2+^, and Cd^2+^, are known to exhibit high toxicity and bioaccumulation effects. These typical heavy metal ions can enter the human and animal body through the food chain, thereby posing serious threats to health [1]. Among them, Hg^2+^ is considered one of the most toxic heavy metal ions, which can lead to various diseases, such as immune dysfunction, kidney disease, and cardiovascular disease, upon accumulation in the human body [2]. The China Food and Drug Administration has stipulated the maximum allowable limit of Hg^2+^ in milk to be 49.9 nM [3]. Therefore, it is of utmost importance to develop a rapid, accurate, and sensitive method for detecting Hg^2+^ to ensure food safety.

Although traditional methods for detecting Hg^2+^, such as atomic absorption spectroscopy (AAS) [4], surface-enhanced Raman scattering (SERS) [5], inductively coupled plasma mass spectrometry (ICP-MS) [6], and atomic fluorescence spectroscopy (AFS) [7] offer high sensitivity and efficiency, they are associated with expensive equipment, well-trained operators, and lengthy preprocessing times [8]. In recent years, many colorimetric [9], fluorescent [10], and electrochemical sensors have been developed as effective tools for Hg^2+^ detection [11]. Among these methods, electrochemical aptasensors are becoming increasingly favored by researchers due to their low consumption, rapidity, stability, and high sensitivity. However, the complex composition and extremely low concentration of actual samples present higher requirements for electrochemical sensors, which need to be addressed in future research.

Recent studies have highlighted the potential of nanocomposite materials to significantly enhance the performance of electrochemical sensors [12]. By combining different materials, nanocomposites can leverage their individual strengths and generate new properties through synergistic effects. Among the various materials being investigated, graphene and graphene-derived materials have emerged as popular areas of study. Graphene aerogels (GAs), a type of graphene-based carbon material, possess a unique three-dimensional network structure that offers highly specific surface area, porosity, conductivity, and low density [13]. GAs can be combined with other materials to form composites with enhanced properties. For instance, Lu et al. [14] synthesized GAs-Ui0-66-NH_2_ by in situ growing metal–organic frameworks (Ui0-66-NH_2_) on GAs. This GAs composite material can simultaneously detect four heavy metal ions, Cd^2+^, Pb^2+^, Cu^2+^, and Hg^2+^, with detection limits of 9 nM, 1 nM, 8 nM, and 0.9 nM, respectively. Similarly, Zhi et al. [15] utilized graphene oxide (GO) doped with molybdenum disulfide (MoS_2_) as the raw material and embedded the Au and Fe_3_O_4_ nanoparticles (NPs) into GO-doped MoS_2_ and prepared porous composite aerogels (CAs) with the hydrothermal method. The CAs not only can sensitively detect mercury ions in aqueous solution with a detection limit of 3.279 nM but also have fast desorption ability.

Aptamers, which are single-stranded DNA or RNA sequences obtained with systematic evolution of ligands with exponential enrichment (SELEX) technology, have the ability to specifically bind to receptors [16]. Due to their low cost, high specificity, high stability, and easy labeling, aptamers have been extensively developed into aptasensors [17]. Ono and Togashi demonstrated that Hg^2+^ can specifically bind two thymine bases (T), forming a stable T-Hg^2+^-T complex [18]. Based on this specific binding, several types of electrochemical aptasensors have been designed to detect Hg^2+^ with high selectivity. For example, Zhang et al. [19] constructed an electrochemical aptasensor for Hg^2+^ detection based on the structure change of a single-stranded DNA rich in T sequences labeled with methylene blue (MB) and specific T-Hg^2+^-T coordination, which has high selectivity and sensitivity with a detection limit of 0.62 fM. Similarly, He et al. [20] developed an electrochemical aptasensor for the ultrasensitive detection of Hg^2+^ using a DNA double-loop signal amplification strategy. In the presence of Hg^2+^, a specific T-Hg^2+^-T was formed, and then exonuclease III (Exo III) triggered the DNA double loop. The aptasensor showed high stability, sensitivity, and good specificity, with a detection limit of 0.19 fM. Wang et al. [21] devised an innovative dual-target electrochemical biosensor with high sensitivity for the detection of Pb^2+^ or Hg^2+^ in water. In the presence of Hg^2+^, it forms a complex structure known as thymidine-Hg^2+^-thymidine (T-Hg^2+^-T) on the aptamer chain, leading to signal attenuation. Conversely, in the presence of Pb^2+^, the DNA single strand S2 dissociates and binds to Pb^2+^, subsequently triggering Exo I to selectively cleave the single strand from the recognition site. This process accomplishes cyclic amplification of the electrochemical signal. The biosensor demonstrates a linear detection range spanning from 100 pg/L to 10.0 mg/L, with detection limits of 82.05 nM for Pb^2+^ and 59.85 nM for Hg^2+^.

The present study employs a triple signal amplification strategy, which offers several advantages over other detection methods. Firstly, the use of a GAs-AuNPs composite as a DNA immobilization platform facilitates electron transfer and enhances the amount of DNA immobilization, thereby reducing the response time and expanding the linear detection range. Secondly, Exo III enzyme-assisted cycling cleaves DNA, leading to an increased amount of DNA helper and a reduced detection limit. Finally, the modification of MB-modified DNA (A1 and A2) into an H-shaped structure enhances the stability of the electrochemical aptasensor, resulting in an amplified electrochemical response signal of MB. These three strategies synergistically contribute to the highly sensitive and selective detection of Hg^2+^. Importantly, the newly fabricated Hg^2+^ aptasensor could also be applied in real milk sample detection.

## 2. Experimental Section

### 2.1. Reagents

Exonuclease III (Exo III) was purchased from Sangon Biotech Co., Ltd. (Shanghai, China). Graphene oxide (GO) was obtained from Xianfeng Nanomaterials Technology Co., Ltd. (Nanjing, China), while 6-mercapto-1-hexanol (MCH, C_6_H_14_OS) was purchased from Aladdin (Shanghai, China). Microporous membrane (Material: Organic Nylon; Diameter: 13 mm; Pore Size: 0.45 μm) was purchased from Taimei Biotechnology Co., Ltd. (Nantong, China). Tetrahydrate chloroauric acid (HAuCl_4_·4H_2_O) was purchased from Nanjing Chemical Reagent Co., Ltd. (Nanjing, China). N, N-dimethylformamide (DMF), and mercury ion standard solution (Hg^2+^) were purchased from Macklin Biochemical Co., Ltd. (Shanghai, China). Sodium citrate (C_6_H_5_Na_3_O_7_·2H_2_O), potassium ferrocyanide (K_3_[Fe(CN)_6_]), potassium ferricyanide (K_4_[Fe(CN)_6_]), potassium chloride (KCl), acetone (C_3_H_6_O), ethanol (C_2_H_6_O), concentrated sulfuric acid (H_2_SO_4_), concentrated hydrochloric acid (HCl), sodium chloride (NaCl), and magnesium chloride (MgCl_2_) were purchased from China National Pharmaceutical Group Chemical Reagent Co., Ltd. (Shanghai, China). All chemicals utilized in this work were of analytical quality and did not necessitate further processing. The aqueous solution employed in the experiment was formulated using distilled water.

### 2.2. Instrumentation

The morphologies and structures of the prepared electrodes were characterized with a scanning electron microscope (SEM, EV0-18, Carl Zeiss AG, Jena, Germany), high-resolution transmission electron microscope (HRTEM, JEM-2100F, JEOL, Tokyo, Japan), Fourier transform infrared spectrometer (FT-IR-850, Tianjin Gangdong Technology Co., Tianjin, China), X-ray Diffractometer (XRD, XD-3, Beijing Pulse Analyzer General Instrument Co., Beijing, China), and Raman spectrometer (XploRA Plus, HORIBA, Kyoto, Japan). All electrochemical measurements were conducted on a CHI 660E electrochemical workstation (Shanghai Chen Hua Instruments Co., Ltd., Shanghai, China) utilizing a standard three-electrode system. This system consisted of a GAs-AuNPs-modified ITO working electrode, a platinum wire counter electrode, and a saturated calomel reference electrode (SCE).

### 2.3. Fabrication of the GAs-AuNPs Sensing Electrode

In order to achieve clean surfaces, the ITO substrates were initially subjected to ultrasonication in acetone, ethanol, and pure water for 10 min each. The electrode area, with a diameter of 4 mm, was then regulated using perforated insulating tape. Afterwards, a solution of 1.0 mg of GAs-AuNPs powder in 1.0 mL of DMF was prepared using ultrasonication to obtain a dispersion of GAs-AuNPs with a concentration of 1.0 mg mL^−1^. Finally, a droplet of 10 μL from the dispersion was applied onto the bare electrode, which was then allowed to air-dry naturally to obtain the GAs-AuNPs/ITO electrode.

### 2.4. Fabrication of the Hg^2+^ Aptasensor

**Scheme 1:**
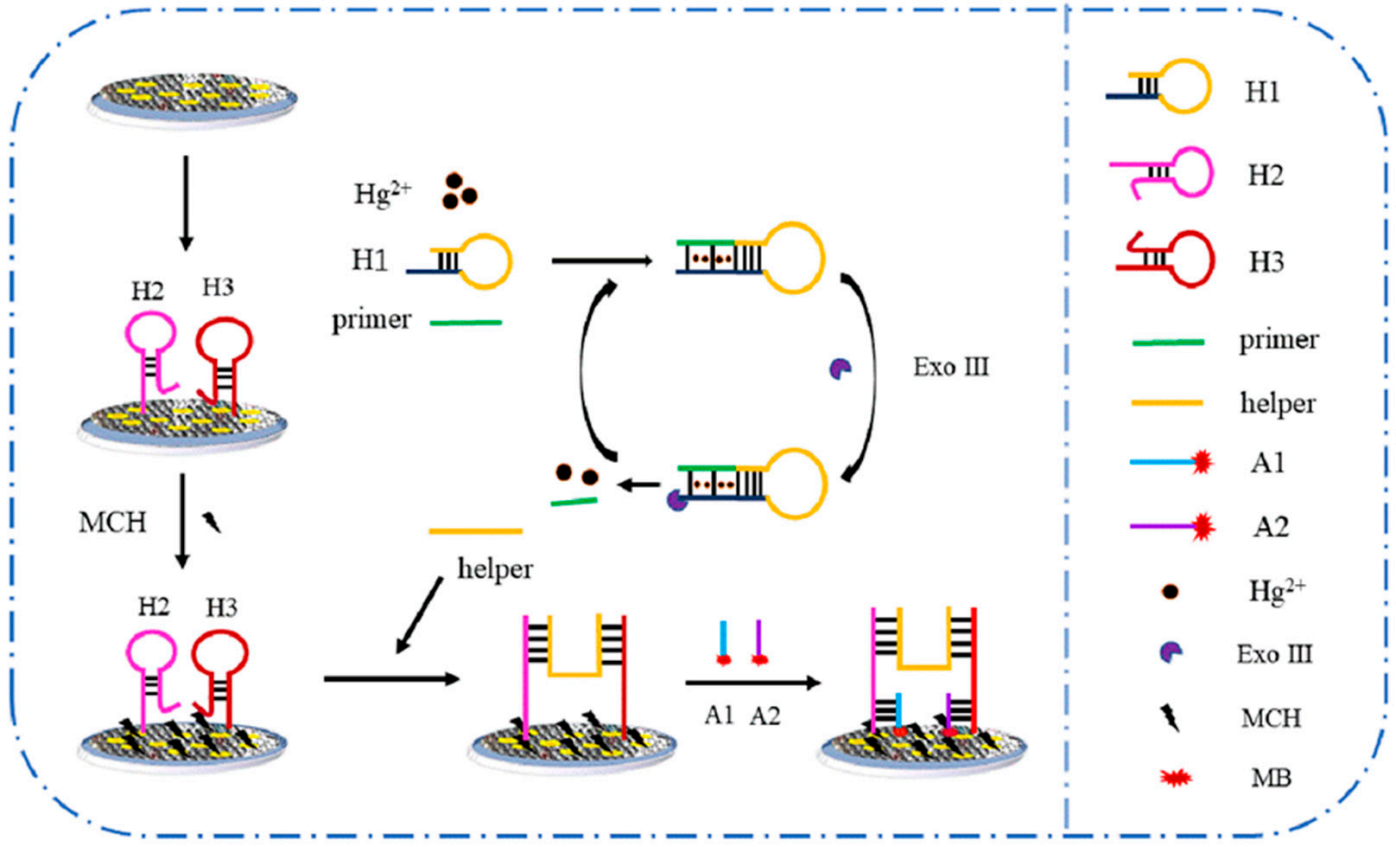
Schematic protocol of the electrochemical Hg^2+^ aptasensor.

All DNA sequences are shown in Table S1. The schematic representation of the Hg^2+^ aptasensor design is illustrated in Scheme 1. Firstly, the thiolated H2 and H3 were covalently immobilized on the GAs-AuNPs electrode surface to form H2-H3/GAs-AuNPs, and then the remaining active sites were effectively passivated with 2 mM MCH for 2 h. In the presence of Hg^2+^, the cycling of Exo III was initiated with the aid of a mixed solution containing H1 (3 μL, 10 μM), primer (3 μL, 5 μM), and Exo III (10 μL, 4 U μL^−1^), resulting in a marked enhancement in the generation of DNA helper. Subsequently, the 10 μL of DNA helper solution obtained above was incubated on the electrode surface to open the hairpin structure of H2 and H3 at 37 °C for 2 h. Finally, 10 μL of a mixture of DNA (A1 and A2) was dropped onto the electrode surface and incubated at 37 °C for 2 h, and the response current of MB was measured using differential pulse voltammetry (DPV).

### 2.5. Pretreatment of Real Samples

To assess the practical utility of the aptasensor developed in this study, we employed it to detect Hg^2+^ in real milk samples. Prior to electrochemical detection, commercially purchased milk underwent pretreatment. Specifically, 1 mL of whole milk was mixed with 9 mL of acetonitrile and shaken for 5 min. The resulting mixture was then subjected to centrifugation at a speed of 8000 r min^−1^ for 10 min, and the supernatant was filtered through a 0.45 μm microporous membrane. The resulting supernatant was subsequently dried in a vacuum oven at 25 °C and reconstituted with 1 mL of distilled water [3]. The milk extract was then analyzed using the standard addition method with varying concentrations of Hg^2+^.

## 3. Results and Discussion

### 3.1. Characterization of Morphology and Composition of GAs-AuNPs

**Figure 1:**
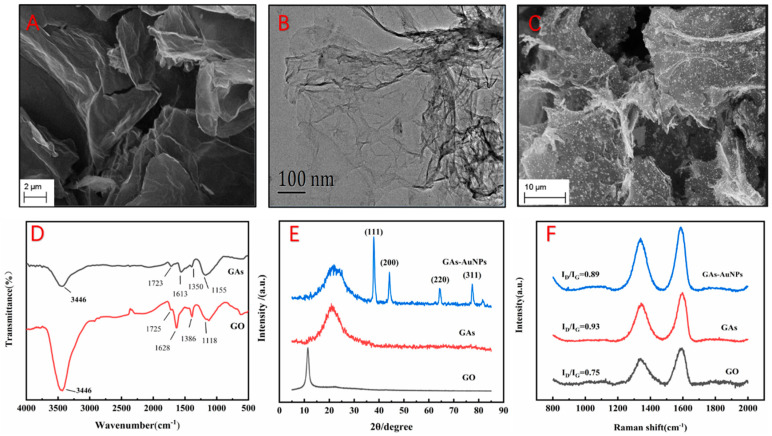
SEM images of (**A**) GAs and (**C**) GAs-AuNPs; TEM image of (**B**) GAs; (**D**) FT-IR spectra of GO and GAs; (**E**) XRD spectrum of GO, GAs, and GAs-AuNPs; (**F**) Raman diagrams of GO, GAs, and GAs-AuNPs.

GAs composites are good candidates for the development of biosensors due to their excellent properties, such as a rich pore structure, large surface area, high conductivity, low density, and good biocompatibility [22,23]. In this study, a GAs-AuNPs-modified electrode was obtained and characterized by different characterization techniques. The morphology and structure of the GAs-AuNPs composite were characterized using SEM and TEM. As shown in Figure 1A, the stacked sheet-like porous structure was clearly observed, and the TEM image in Figure 1B shows a wrinkled and cross-linked structure, indicating that a GAs was successfully synthesized [14]. The SEM image of the GAs-AuNPs composite is shown in Figure 1C, which indicates the uniform distribution of AuNPs on the surface of the GAs.

The chemical structures of GO and GA samples were also analyzed with FT-IR. As shown in Figure 1D, the absorption peaks at 3446 cm^−1^, 1725 cm^−1^, 1628 cm^−1^, 1386 cm^−1^, and 1118 cm^−1^ correspond to the stretching vibration of the -OH functional group, the stretching vibration of the -C=O functional group, the stretching vibration of the -C=C functional group, the stretching vibration of the -C-O functional group, and the stretching vibration of the -O- functional group in GO, respectively. In the FT-IR spectra of GAs, the intensities of the functional groups (-OH, -C=O, -C=C, -C-O, and -O-) are significantly reduced, indicating that the graphene aerogel prepared with the hydrothermal method formed a network of interconnected porous structures [24,25,26].

Figure 1E shows the XRD patterns of the samples GO, GAs, and GAs-AuNPs. The GO curve has a sharp diffraction peak at 11.3°, which is the graphene (002) plane. The GAs curve has a broad and relatively sharp diffraction peak at 22.04°, which indicates the reduction in GO [25,27]. According to the JCPDS (NO.04-0784), the XRD pattern of the GAs-AuNPs composite has four diffraction peaks at 37.8°, 44.1°, 64.5°, and 77.6° corresponding to the (111), (200), (220), and (311) crystal faces of the AuNPs, respectively. Meanwhile, the diffraction peak of the (111) plane of the AuNPs is significantly higher than the other peaks [28,29].

Similarly, the Raman spectra of the samples of GO, GAs, and GAs-AuNPs are shown in Figure 1F. The D band represents the degree of disorder in the carbon material, and the G band represents the degree of sp^2^ hybridization. The degree of defects in the carbon material can be represented by the intensity ratio of the D band to the G band (ID/IG) [30]. Each curve has two obvious peaks at 1345.2 cm^−1^ and 1593.2 cm^−1^, corresponding to the D band and the G band, respectively. The larger the ID/IG ratio, the greater the defects in graphene [31]. The ID/IG of GO is 0.75, and the ID/IG of GAs is 0.93, indicating the disappearance of the large sp^2^ plane in the surface carbon skeleton and the formation of a large number of graphene sheets [32]. However, the ID/IG of GAs-AuNPs is 0.89, which is lower than that of GAs, indicating that the introduction of AuNPs led to a change in the sp^2^ carbon network [33].

### 3.2. Electrochemical Characterization of the Hg^2+^ Aptasensor

**Figure 2:**
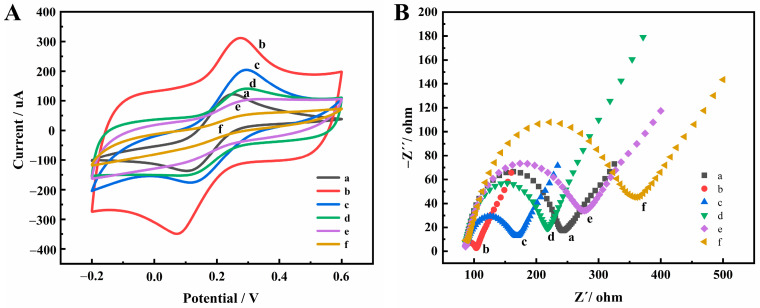
CV (**A**) and EIS spectra (**B**) at different modification steps: (a) bare electrode, (b) GAs-AuNPs, (c) H2-H3/GAs-AuNPs, (d) MCH/H2-H3/Gas-AuNPs, (e) helper/MCH/H2−H3/GAs-AuNPs, (f) A1-A2/helper/MCH/H2-H3/GAs-AuNPs.

The influence of the preparation method of the GAs-AuNPs composites on their conductivity was investigated, and the electrochemical properties of the GAs-AuNPs-modified electrode were studied, as detailed in the Supplementary Materials. The process of constructing the electrochemical aptasensor was characterized using CV and EIS. Figure 2A shows the CV curves of the bare electrode (curve a) and the electrodes after different modification steps: the GAs-AuNPs-modified electrode (curve b). H2-H3/GAs-AuNPs-modified electrode (curve c), passivated with MCH (curve d), helper/H2-H3/GAs-AuNPs-modified electrode (curve e), and A1-A2/helper/H2-H3/GAs-AuNPs-modified electrode (curve f). A pair of well-defined redox peaks was observed for the GAs-AuNPs-modified electrode. When H2 and H3 were immobilized onto the electrode surface, the peak current decreased significantly. This is due to the electrostatic repulsion of the DNA on the electrode surface, which has a large number of negatively charged phosphate groups, against [Fe(CN)_6_]^3−/4−^ [34]. Subsequently, the nonspecific binding sites were blocked with MCH, and the response current continued to decrease. In the presence of Hg^2+^, DNA strand helper was released via the Exo III-mediated cyclic amplification process. The electrode was further modified with the obtained DNA strand helper, which was bound to the electrode surface with H2 and H3 through complementary base pairing, further reducing the response current. Finally, hybridization with A1 and A2 further reduced the response current.

Meanwhile, the modification process was assessed using Electrochemical Impedance Spectroscopy (EIS), as depicted in Figure 2B. Initially, the bare electrode (curve a) exhibited the interfacial charge-transfer resistance (*R_ct_*) value of 156 Ω, indicating relatively sluggish electron transfer on its surface. However, upon modification with GAs and AuNPs (curve b), the *R_ct_* value significantly decreased to 17 Ω, suggesting enhanced electron transfer due to the presence of GAs and AuNPs. Subsequently, the electrode surface was immobilized with H2 and H3 (curve c), leading to an increased *R_ct_* value of 84 Ω. This elevation can be attributed to the electrostatic repulsion between the negatively charged oligonucleotides and the negative [Fe(CN)_6_]^3−/4−^ probe. To determine the fractional surface coverage (*θ*) of H2 and H3 on the electrode, the formula *θ* = 1 − (*R_ct(b)_*/*R_ct(c)_*) × 100% was employed, where *R_ct(b)_* and *R_ct(c)_* represented the *R_ct_* values of the bare GAs-AuNPs electrode and the H2- and H3-modified electrode, respectively. Consequently, a *θ* value of 80% was calculated, signifying a relatively high coverage of H2 and H3 [35]. Further modifications were performed sequentially on the electrode, involving the addition of MCH (curve d), helper (curve e), and A1 and A2 (curve f). These subsequent modifications caused an incremental increase in *R_ct_* values to 132 Ω, 190 Ω, and 274 Ω, respectively. The observed rise in *R_ct_* values indicates the successful construction of the Hg^2+^ aptasensor. In summary, the characterization of the modification process using EIS demonstrated the progressive transformation of the electrode, with improved electron transfer upon the incorporation of GAs and AuNPs. The immobilization of H2 and H3, followed by the subsequent modifications, resulted in elevated *R_ct_* values, ultimately confirming the successful construction of the Hg^2+^ aptasensor.

### 3.3. Signal Amplification Using H-Shaped Structure

**Figure 3:**
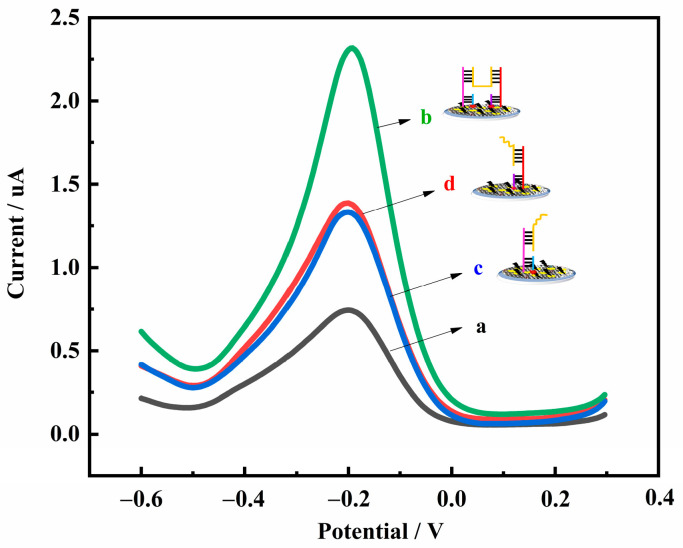
DPV diagrams obtained with the electrode modified with different conditions: (a) without H2 and H3; (b) with H2 and H3; (c) with H2 only; (d) with H3 only.

In order to improve the sensitivity of the detection method, a signal amplification process using the H-shaped structure was applied. A comparison was made between the electrode modified with H2 and H3, H2 only, and H3 only. When 1 pM Hg^2+^ was added to the system, different DPV plots were obtained after different procedures, as shown in Figure 3. The current responses of MB of the H2- and H3-modified electrode (curve b), the H2-modified electrode (curve c), and the H3-modified electrode (curve d) were 2.321, 1.365, and 1.371 μA, respectively. These results strongly suggest that the H-shaped structure caused a 1.7-fold increase in current compared to the electrode modified with H2 or H3.

### 3.4. Optimization of Experimental Conditions

To ensure optimal detection performance for Hg^2+^, we optimized several experimental parameters for the prepared sensing platform. Specifically, we focused on the amount of GAs-AuNPs, the dosage of the mixture of H2 and H3, the Exo III digestion time, and the dosage of DNA primer.

**Figure 4:**
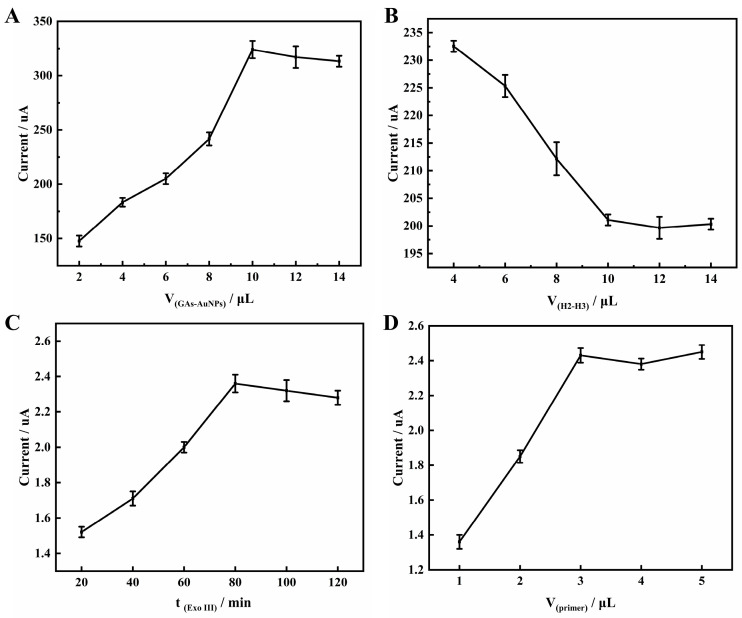
Effects of (**A**) GAs-AuNPs dosage and (**B**) H2 and H3 mixture dosage on the CV peak in 5 mM [Fe(CN)_6_]^3−/4−^ containing 0.1 M KCl. Effects of (**C**) Exo III enzyme digestion time and (**D**) DNA strand primer dosage on the DPV peak of aptasensor in PBS (pH 7.4).

The amount of GAs-AuNPs on the electrode is a critical factor that directly affects the performance of the electrochemical sensor. In this study, the concentration of GAs-AuNPs was fixed at 1 mg mL^−1^. As shown in Figure 4A, the response current of the electrode to [Fe(CN)_6_]^3−/4−^ gradually increased as the droplet dosage of GAs-AuNPs ranged from 2 μL to 10 μL. This increase was due to the introduction of GAs-AuNPs, which accelerated the transfer of electrons on the electrode surface. As the dosage was further increased, the response current remained stable owing to the tendency of the thicker GAs-AuNPs film to drop off [36]. Therefore, from an economic point of view, we chose 10 μL as the optimal amount of GAs-AuNPs in this work.

The dosage of H2 and H3 immobilized on the surface of GAs-AuNPs as capture probes can significantly affect the performance of the Hg^2+^ aptasensor, as the construction of the sensing interface is crucial. Therefore, optimizing the amount of H2 and H3 is necessary. At a fixed concentration of 5 μM for the mixture of H2 and H3, it was observed from the curve in Figure 4B that as the mixture dosage increased from 4 to 10 μL, the peak current of [Fe(CN)_6_]^3−/4−^ decreased. This was due to the negatively charged oligonucleotide phosphate backbone, which prevented [Fe(CN)_6_]^3−/4−^ from approaching the electrode surface. The peak current then stabilized, indicating the saturation of H2 and H3 immobilization. Therefore, 10 μL of the mixture of H2 and H3 dosage was sufficient for the subsequent experiments.

The digestion time of Exo III directly affects the detection limit of electrochemical sensors. In order to achieve a more thorough hydrolysis of double-stranded DNA, an amount of 10 μL of Exo III at a concentration of 4 U μL^−1^ was chosen in this study. The optimization curve of Exo III digestion time is presented in Figure 4C. The peak current demonstrated an increase as the digestion time was prolonged from 20 to 80 min, after which it stabilized. Therefore, 80 min was accepted as the optimum digestion time.

The DNA primer plays a crucial role in the Exo III cycling system of the sensor, as it determines the ability to form T-Hg^2+^-T with H1 and Hg^2+^. Thus, optimizing the amount of primer is essential. In this work, a concentration of 5 μM was used, and the optimization curve of the dosage of DNA chain primer was presented. As shown in Figure 4D, with the amount of primer increasing from 1 to 3 μL, the response current of MB gradually increased. However, when the amount of primer continued to increase, the response current almost stabilized, indicating that the reaction between the primer and H1 in the solution had reached saturation level. Therefore, 3 μL was selected as the optimal amount.

### 3.5. Detection Performances of Hg^2+^ Electrochemical Sensor

**Figure 5:**
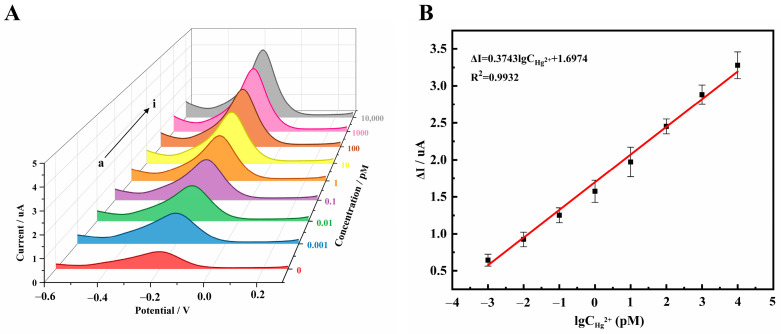
(**A**) DPV diagrams of the aptamer sensor for different concentrations of Hg^2+^: (a) 0, (b) 1 fM, (c) 10 fM, (d) 0.1 pM, (e) 1 pM, (f) 10 pM, (g) 0.1 nM, (h) 1 nM, (i) 10 nM; (**B**) The linear fitting diagram of the change value of DPV peak current and the log value of Hg^2+^ concentration.

The DPV curves were obtained under optimized conditions, and the results showed that the DPV peak current increased as the concentration of Hg^2+^ increased from 0 fM to 10 nM, as shown in Figure 5A. Meanwhile, a linear relationship between the value of peak current changes (Δ*I*) and the logarithm of *C_Hg_*_2+_ (*lgC_Hg_*^2+^) ranging from 1 fM to 10 nM is shown in Figure 5B. The linear regression equation is Δ*I* = 0.3743 *lgC_Hg_*^2+^ + 1.6974 (R^2^ = 0.9932), with a limit of detection (LOD) of 0.16 fM (LOD = 3$\sqrt{\frac{\sum_{i=1}^{m}{\left(I_{0i}-\bar{I_{0}}\right)}^{2}}{m-1}}$/0.3743, *I*_0_ is the signal of blank sample, *m* = 5). The method presented herein exhibits a detection limit seven orders of magnitude lower than the inductively coupled plasma mass spectrometry utilized in the National Standard of the People’s Republic of China [37]. This indicates our ability to detect mercury ions at far lower concentrations, an achievement attributable to the triple amplification design of this strategy. Moreover, our method also meets the requirements of the China Food and Drug Administration for the limit of Hg^2+^ in food. To further substantiate the superiority of our work, we performed a comparative analysis of the linear detection range and detection limit of the aptasensor as reported in other literature. From Table 1, it can be seen that our method has a lower detection limit compared to other detection methods and meets higher standard requirements, indicating that our sensor has promising prospects for applications.

### 3.6. Specificity, Reproducibility, and Stability of the Electrochemical Biosensor

In order to assess the feasibility of the proposed method, we conducted a comprehensive investigation into the specificity, reproducibility, and stability of the aptasensor. To evaluate the specificity of this aptasensor, we examined the potential interference of other metal ions, including Mg^2+^, Pb^2+^, Ca^2+^, Na^+^, K^+^, Cd^2+^, and Cr^6+^. The evaluation was carried out by recording the current response (*Ip*) induced with blank or other metal ions and Hg^2+^. As shown in Figure S3A, the *Ip* generated with nontarget metal ions (2 mM) was comparable to that of the blank. In contrast, the introduction of 1 pM Hg^2+^ resulted in a distinct *Ip*. Notably, the coexistence of other metal ions with Hg^2+^ did not significantly affect the *Ip*, as compared to that of Hg^2+^ alone. These results suggest that the proposed electrochemical sensing platform exhibits high specificity for the detection of Hg^2+^ in the presence of multiple metal ions. Reproducibility tests were conducted on three batches of five independent aptasensors to detect 1 pM Hg^2+^. The detection results had a relative standard deviation (RSD) value of 2.75% (Figure S3B). The aptasensor demonstrated good reproducibility due to the precise control of experimental conditions during fabrication. Furthermore, the stability of the aptasensor was evaluated by analyzing Hg^2+^. One aptasensor was fabricated and stored at 4 °C for 15 days. The peak current response of this aptasensor to 1 pM Hg^2+^ was found to retain 91.1% of its initial response (Figure S3C), indicating excellent stability of the proposed aptasensor. This can be attributed to the mechanical stiffness and structural stability of the H-shaped structure and the good biocompatibility of GAs-AuNPs to stabilize the activity of biomolecules.

### 3.7. Real Sample Detection

To confirm the feasibility and applicability of the sensor, we tested different concentrations of Hg^2+^ in milk samples using a standard addition method. It can be seen in Table 2 that the recovery rate of sensor samples is from 97.3% to 105.3%, indicating that this method has potential practical value.

## 4. Conclusions

In this study, we successfully developed a highly sensitive, selective, and stable electrochemical aptasensor for the detection of Hg^2+^. This study had two main highlights: (1) GAs-AuNPs film possessed such advantages as good conductivity, desirable biocompatibility, abundant pore structure, and large surface area, forming a high-performance sensing interface, which can stabilize more DNA (H2 and H3) and accelerate electronic transfer between the electrode surface and the electroactive probe; (2) Exo III and H-shaped structures were used to improve sensitivity by mediating the cyclic amplification process. Under the optimal conditions, the LOD of the Hg^2+^ detection was down to 0.16 fM. Meanwhile, the aptasensor was used to detect real milk samples with good results. More importantly, the analysis presented showcases a universal approach to monitoring various metal ions in food matrices on-site by employing distinct aptamer traps.

## Figures and Tables

**Table 1:** Comparison of performance of several aptasensors for detecting Hg^2+^.

Methods	Linear Range	LOD	References
SERS	0.01 μM–0.1 mM	9.83 nM	[38]
Fluorescence	0–500 nM	9.2 nM	[39]
Luminescence	0–0.03 M	48 μM	[40]
Fluorescence	0–40 μM	0.12 μM	[41]
Electrochemical	1 fM–10 nM	0.19 fM	[20]
Electrochemical	0.2–2000 pM	0.12 pM	[42]
Electrochemical	1 fM–10 nM	0.16 fM	This work

**Table 2:** Determination of Hg^2+^ in milk samples with the proposed aptasensor.

Sample	No.	Added	Proposed	Recovery	RSD
		(nM)	Aptasensor (nM)	(%)	(*n* = 3, %)
	1	0.3	0.292	97.3	3.37
milk	2	1.0	0.978	97.8	2.68
	3	2.5	2.633	105.3	1.41

## Data Availability

The data presented in this study can be obtained from the first author.

## Supplementary Materials

The following supporting information can be downloaded at: https://www.mdpi.com/article/10.3390/bios13100932/s1, Oligonucleotides; Table S1: DNA sequences; Electrochemical measurement; Synthesis of GAs-AuNPs composite; Characterization of the electrochemical properties of the GAs-AuNPs; Figure S1: CV (A) and EIS spectra (B) at different modification steps: (a) bare electrode, (b) AuNPs-modified electrode, (c) GAs-modified electrode, and (d) GAs-AuNPs-modified electrode; Figure S2: CVs (A) and EIS spectra (B) at different preparation methods: (a) bare electrode, (b) modified electrode with method 1, (c) modified electrode with method 2, and (d) modified electrode with method 3; Figure S3: (A) The selectivity of the aptamer sensor to Mg^2+^, Pb^2+^, Ca^2+^, Na^+^, K^+^, Cd^2+^, Cr^6+^, blank concentration, seven kinds of interfering ions without Hg^2+^, and seven kinds of interfering ions with Hg^2+^ and only Hg^2+^ (the concentration of Hg^2+^ is 1 pM, others are all 2 mM); (B) Repeatability of 5 different electrodes modified with 1 pM Hg^2+^; (C) Stability of the aptasensor at 0, 5, 10, and 15 days [43,44,45].

## References

[1] Chen Z., Zhang Z., Qi J., You J., Ma J., Chen L. (2023). Colorimetric detection of heavy metal ions with various chromogenic materials: Strategies and applications. J. Hazard. Mater..

[2] Yin K., Lv M., Wang Q., Wu Y., Liao C., Zhang W., Chen L. (2016). Simultaneous bioremediation and biodetection of mercury ion through surface display of carboxylesterase E2 from Pseudomonas aeruginosa PA1. Water Res..

[3] Zhang M., Guo W. (2023). Simultaneous electrochemical detection of multiple heavy metal ions in milk based on silica-modified magnetic nanoparticles. Food Chem..

[4] Souza J.P., Cerveira C., Miceli T.M., Moraes D.P., Mesko M.F., Pereira J.S.F. (2020). Evaluation of sample preparation methods for cereal digestion for subsequent As, Cd, Hg and Pb determination by M-based techniques. Food Chem..

[5] Tangsuwanjinda S., Chen Y.Y., Lai C.H., Jhou G.T., Chiang Y.W., Cheng H.M. (2021). Microporous oxide-based surface-enhanced Raman scattering film for quadrillionth detection of mercury ion (II). Processes.

[6] Sun C., Zhang Y., Gong Z., Wang X., Yang Y., Wang Y. (2018). Determination of trace elements in samples with high salt content by inductively coupled plasma mass spectrometry after solid-phase preconcentration. Int. J. Mass Spectrom..

[7] Sorouraddin S.M., Farajzadeh M.A., Dastoori H. (2020). Development of a dispersive liquid-liquid microextraction method based on a ternary deep eutectic solvent as chelating agent and extraction solvent for preconcentration of heavy metals from milk samples. Talanta.

[8] Che S., Yin L., Chen M., Fan Y., Xu A., Zhou C., Fu H., She Y. (2023). Real-time monitoring of mercury (II) in water and food samples using a quinoline-based ionic probe. Food Chem..

[9] Khani H., Abbasi S., Yaraki M.T., Tan Y.N. (2022). A naked-eye colorimetric assay for detection of Hg^2+^ ions in real water samples based on gold nanoparticles-catalyzed clock reaction. J. Mol. Liq..

[10] Pan Y., Guo Y., Li Y., Tang L., Yan X. (2023). A new aggregation-induced emission-based fluorescent probe for effective detection of Hg^2+^ and its multiple applications. Chin. Chem. Lett..

[11] Zhang Y., Xu Y., Li N., Liu X., Ma Y., Luo H., Hou C., Huo D. (2023). An ultrasensitive electrochemical sensor based on antimonene simultaneously detect multiple heavy metal ions in food samples. Food Chem..

[12] Reddy Y.V.M., Shin J.H., Palakollu V.N., Sravani B., Choi C.H., Park K., Kim S.K., Madhavi G., Park J.P., Shetti N.P. (2022). Strategies, advances, and challenges associated with the use of graphene-based nanocomposites for electrochemical biosensors. Adv. Colloid Interfac..

[13] Yang J., Li Y., Zheng Y., Xu Y., Zheng Z., Chen X., Liu W. (2019). Versatile aerogels for sensors. Small.

[14] Lu M., Deng Y., Luo Y., Lv J., Li T., Xu J., Chen S.W., Wang J. (2019). Graphene aerogel-metal-organic framework-based electrochemical method for simultaneous detection of multiple heavy-metal ions. Anal. Chem..

[15] Zhi L., Zuo W., Chen F., Wang B. (2016). 3D MoS_2_ composition aerogels as chemosensors and adsorbents for colorimetric detection and high-capacity adsorption of Hg^2+^. ACS Sustain. Chem. Eng..

[16] Tuerk C., Gold L. (1990). Systematic evolution of ligands by exponential enrichment: RNA ligands to bacteriophage T4 DNA polymerase. Science.

[17] Guo W., Zhang C., Ma T., Liu X., Chen Z., Li S., Deng Y. (2021). Advances in aptamer screening and aptasensors’ detection of heavy metal ions. J. Nanobiotechnol..

[18] Ono A., Togashi H. (2004). Highly selective oligonucleotide-based sensor for mercury (II) in aqueous solutions. Angew. Chem. Int. Ed..

[19] Zhang X., Huang C., Jiang Y., Jiang Y., Shen J., Han E. (2018). Structure-switching electrochemical aptasensor for single-step and specific detection of trace mercury in dairy products. J. Agric. Food Chem..

[20] He W., Qiao B., Li F., Pan L., Chen D., Cao Y., Tu J., Wang X., Lv C., Wu Q. (2021). A novel electrochemical biosensor for ultrasensitive Hg^2+^ detection via a triple signal amplification strategy. Chem. Commun..

[21] Wang Y., Zhai H., Yin J., Guo Q., Zhang Y., Yang Q., Li F., Sun X., Guo Y., Zhang Y. (2022). Dual-target electrochemical DNA sensor for detection of Pb^2+^ and Hg^2+^ simultaneously by exonuclease I–assisted recycling signal amplification. Microchim. Acta.

[22] Kim M.Y., Seo K.D., Park H., Mahmudunnabi R.G., Lee K.H., Shim Y.B. (2022). Graphene-anchored conductive polymer aerogel composite for the electrocatalytic detection of hydrogen peroxide and bisphenol A. Appl. Surf. Sci..

[23] Li Z., Liu C., Frick J.J., Davey A.K., Dods M.N., Carraro C., Senesky D.G., Maboudian R. (2023). Synthesis and characterization of UiO-66-NH_2_ incorporated graphene aerogel composites and their utilization for absorption of organic liquids. Carbon.

[24] Liu C., Liu H., Xu A., Tang K., Huang Y., Lu C. (2017). In situ reduced and assembled three-dimensional graphene aerogel for efficient dye removal. J. Alloys Compd..

[25] Trinh T.T.P.N.X., Giang N.T.H., Thinh D.B., Dat N.M., Trinh D.N., Hai N.D., Oanh D.T.Y., Nam H.M., Phong M.T., Hieu N.H. (2022). Hydrothermal synthesis of titanium dioxide/graphene aerogel for photodegradation of methylene blue in aqueous solution. J. Sci. Adv. Mater. Dev..

[26] Tu T.H., Cam P.T.N., Phong M.T., Nam H.M., Hieu N.H. (2019). Synthesis and application of graphene oxide aerogel as an adsorbent for removal of dyes from water. Mater. Lett..

[27] Wang Y., Jin Y., Zhao C., Pan E., Jia M. (2018). Fe_3_O_4_ nanoparticle/graphene aerogel composite with enhanced lithium storage performance. Appl. Surf. Sci..

[28] Ren Z., Li H., Li J., Cai J., Zhong L., Ma Y., Pang Y. (2023). Green synthesis of reduced graphene oxide/chitosan/gold nanoparticles composites and their catalytic activity for reduction of 4-nitrophenol. Int. J. Biol. Macromol..

[29] Yu Y., Wang X., Li M., Liu D. (2023). Design fabrication of electrochemical sensor based on Ru(bpy)_2_^2+^/SMWCNTs/Au/GCE electrode for the selective determination of 5′-guanosine monophosphate. Food Chem..

[30] Sang S., Li D., Zhang H., Sun Y., Jian A., Zhang Q., Zhang W. (2017). Facile synthesis of AgNPs on reduced graphene oxide for highly sensitive simultaneous detection of heavy metal ions. RSC Adv..

[31] Li M., Zhe T., Li R., Bai F., Jia P., Xu Z., Wang X., Bu T., Wu H., Wang L. (2023). ZIF-derived Co nanoparticles embedded into N-doped carbon nanotube composites for highly efficient electrochemical detection of nitrofurantoin in food. Food Chem..

[32] Chu H., Zhang F., Pei L., Cui Z., Shen J., Ye M. (2018). Ni, Co and Mn doped SnS_2_-graphene aerogels for supercapacitors. J. Alloys Compd..

[33] Ma X., Chen D., Tu X., Gao F., Xie Y., Dai R., Lu L., Wang X., Qu F., Yu Y. (2019). Ratiometric electrochemical sensor for sensitive detection of sunset yellow based on three-dimensional polyethyleneimine functionalized reduced graphene oxide aerogels@ Au nanoparticles/SH-β-cyclodextrin. Nanotechnology.

[34] Li X., Peng G., Cui F., Qiu Q., Chen X., Huang H. (2018). Double determination of long noncoding RNAs from lung cancer via multi-amplified electrochemical genosensor at sub-femtomole level. Biosens. Bioelectron..

[35] Peng G., Li X., Cui F., Qiu Q., Chen X., Huang H. (2018). Aflatoxin B1 electrochemical aptasensor based on tetrahedral DNA nanostructures functionalized three dimensionally ordered macroporous MoS_2_-AuNPs film. ACS Appl. Mater. Interfaces.

[36] Peng G., Yu Y., Chen X., Huang H. (2020). Highly sensitive amperometric α-ketoglutarate biosensor based on reduced graphene oxide-gold nanocomposites. Int. J. Anal. Chem..

[37] (2016). Determination of Multiple Elements in Foods. National Standard for Food Safety.

[38] Guo N., Xu G., Zhang Q., Song P., Xia L. (2022). AgNPs functionalized with dithizone for the detection of Hg^2+^ based on surface-enhanced Raman scattering spectroscopy. Plasmonics.

[39] Liu K., Xia C., Guo Y., Yu H., Xie Y., Yao W. (2023). Polyethylenimine-functionalized nitrogen and sulfur co-doped carbon dots as effective fluorescent probes for detection of Hg^2+^ ions. Spectrochim. Acta A.

[40] Li Y., Ma D., Chen C., Chen M., Li Z., Wu Y., Zhu S., Peng G. (2019). A hydrostable and bromine-functionalized manganese-organic framework with luminescence sensing of Hg^2+^ and antiferromagnetic properties. J. Solid State Chem..

[41] Li H., Li J., Pan Z., Zheng T., Song Y., Zhang J., Xiao Z. (2023). Highly selective and sensitive detection of Hg^2+^ by a novel fluorescent probe with dual recognition sites. Spectrochim. Acta A.

[42] Zhong Y.Q., Ning T.J., Cheng L., Xiong W., Wei G.B., Liao F.S., Ma G.Q., Hong N., Cui H.F., Fan H. (2021). An electrochemical Hg^2+^ sensor based on signal amplification strategy of target recycling. Talanta.

[43] Chen Y., Zhao P., Hu Z., Liang Y., Han H., Yang M., Luo X., Hou C., Huo D. (2023). Amino-functionalized multilayer Ti_3_C_2_T_x_ enabled electrochemical sensor for simultaneous determination of Cd^2+^ and Pb^2+^ in food samples. Food Chem..

[44] Karaman C. (2021). Orange peel derived-nitrogen and sulfur Co-doped carbon dots: A nano-booster for enhancing ORR electrocatalytic performance of 3D graphene networks. Electroanalysis.

[45] Zhang Z., Karimi-Maleh H. (2023). In situ synthesis of label-free electrochemical aptasensor-based sandwich-like AuNPs/PPy/Ti_3_C_2_T_x_ for ultrasensitive detection of lead ions as hazardous pollutants in environmental fluids. Chemosphere.

