# Supplementary material for: Development of a Novel H-Shaped Electrochemical Aptasensor for Detection of Hg2+ Based on Graphene Aerogels–Au Nanoparticles Composite
Source: Biosensors (Basel). 2023 Oct 18;13(10):932. doi: 10.3390/bios13100932 (PMC10605725; doi:10.3390/bios13100932)
Supplement: Supplementary file 1 [file biosensors-13-00932-s001.zip › biosensors-2604118-supplementary.pdf]

biosensors

MDPI

Supplementary Material

Development of a Novel H-Shaped Electrochemical Aptasensor for Detection of Hg^{2+} Based on Graphene Aerogels–Au Nanoparticles Composite

Gang Peng *, Mengxue Guo, Yuting Liu, Han Yang, Zuorui Wen and Xiaojun Chen

1. Experimental Section

1.1. Oligonucleotides

All oligonucleotides used in this study were procured from Sangon Biotech Co., Ltd. (Shanghai, China). Sequences of the synthesized oligonucleotides are listed in Table S1.

Table S1. DNA sequences.

Name	Sequences (5'-3')
primer	CTTTCTTCTTT
H1	GCGTTTGTATGTTACTGCTGGCCCGGTCTATAGCAGTAACATACAAACGCTTTG TTGTTTG
H2	SH-C_6-TTCTTTGTATGCCGATCCTAACATACAAACGC
H3	ATAGACCGGGCCAGGAATAATGCCCGGTCCGC-C_6-HS
helper	GCGTTTGTATGTTACTGCTGGCCCGGTCTAT
A1	CATACAAA-MB
A2	MB-GACCGGGC

1.2. Electrochemical Measurement

All the DPV data were recorded in PBS (containing 100 mM NaCl, 5 mM KCl, 1 mM $MgCl_2$, pH 7.4) at room temperature. DPV was recorded within the potential range from 0.3 to −0.6 V under a modulation amplitude of 50 mV and a scan rate of 50 mV s^{-1}; CV was performed within a 0.1 M KCl and 5 mM $[Fe(CN)_6]^{3-/4-}$ at a scan rate of 0.1 mV s^{-1}, scanning potential −0.2 ~ +0.6 V; EIS was performed within 0.1 M KCl aqueous solution containing 5 mM $[Fe(CN)_6]^{3-/4-}$ in the frequency range from 100 kHz to 0.01 Hz with an amplitude of 0.005 V.

1.3. Synthesis of GAs-AuNPs composite

The synthesis of GAs-AuNPs composite material involved a series of steps. Firstly, 40 mg of graphene oxide was accurately weighed and added to 15 mL of deionized water, followed by sonication for 1 h. Subsequently, 4.0 mL of $HAuCl_4 \cdot 4H_2O$ (1mg mL^{-1}) solution was mixed with the graphene oxide solution, and 3 mL of 1% sodium citrate was added, followed by sonication for 30 min. The mixed solution was then transferred to a 100 mL poly tetrafluoroethylene reaction kettle and heated at 180 ℃ for 12 h. After natural cooling to room temperature, the solution was prefrozen for 2 h in a low-temperature freezer (-40 ℃) and finally freeze-dried in a vacuum freeze dryer (-55 ℃, 12 h) to obtain a porous structure of GAs-AuNPs composite material.

2. Results and Discussion

2.1. Characterization of the Electrochemical Properties of the GAs-AuNPs

To investigate the changes in the electrochemical properties of the modified electrodes, CV and EIS techniques were used to study the electrode modification process. Figure S1A shows the CV curves of the bare electrode (curve a), AuNPs-modified electrode

(curve b), GAs-modified electrode (curve c), and GAs-AuNPs-modified electrode (curve d). The current response of the GAs-AuNPs-modified electrode was the highest among all the modified electrodes due to the synergy of GAs and AuNPs based on the large specific surface area of GAs and the excellent conductivity of AuNPs and GAs. According to the Randled-Sevcik formula (1) [1]:

$$I_p=2.69\times10^5 n^{3/2} A D^{1/2} \nu^{1/2} C \quad (1)$$

where I_p is the peak current, n is the number of electronic transfers (=1), D shows the diffusion coefficient (6.7×10^{-6} cm^2 s^{-1}), ν is the scan rate (0.1 V s^{-1}), and the concentration of $[Fe(CN)_6]^{3-/4-}$ is C (5×10^{-6} mol/cm^3). By calculating the data, the effective surface area of bare electrode, AuNPs-modified electrode, GAs-modified electrode, and GAs-AuNPs-modified electrode were 0.111 cm^2, 0.155 cm^2, 0.213 cm^2, and 0.283 cm^2, respectively. Compared with the bare electrode, AuNPs-modified electrode, and GAs-modified electrode, the effective surface area of the GAs-AuNPs-modified electrode increased 2.55-fold, 1.83-fold, and 1.33-fold, respectively.

Simultaneously, we performed EIS characterization, and the results are shown in Figure S1B. The R_{ct} value of the bare electrode (curve a), AuNPs-modified electrode (curve b), GAs-modified electrode (curve c), and GAs-AuNPs-modified electrode (curve d) were 157 Ω, 83 Ω, 40 Ω, and 17 Ω, respectively. Similarly, the R_{ct} value was the smallest after the electrode was modified with GAs-AuNPs, indicating that it can greatly promote the transfer of electrons on the electrode surface.

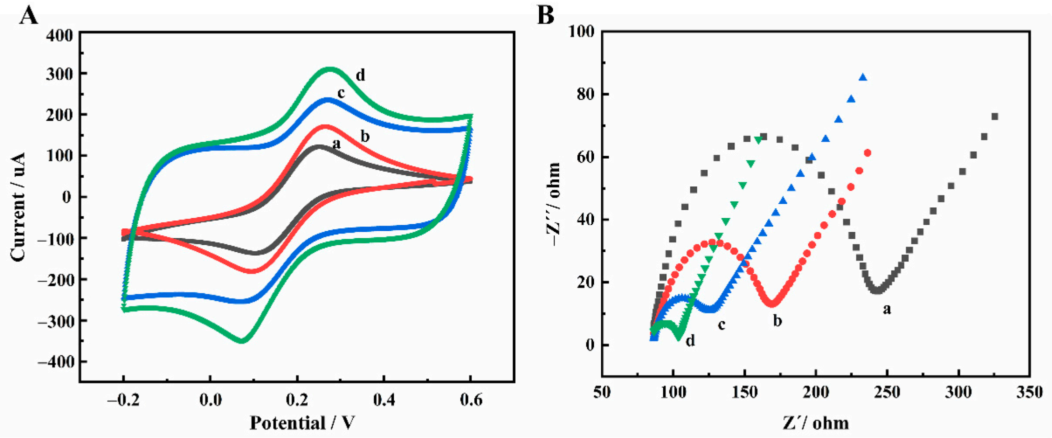

Figure S1. CV (A) and EIS spectra (B) at different modification steps: (a) bare electrode, (b) AuNPs-modified electrode, (c) GAs-modified electrode, and (d) GAs-AuNPs-modified electrode.

In order to investigate the influence of the preparation method of GAs-AuNPs composites on their conductivity, we conducted a comparative analysis of three experimental methods. Method 1 involved the direct drop coating of a mixture of GAs and AuNPs, which were separately prepared using hydrothermal synthesis. Method 2 involved the drop coating of GAs, followed by the electrodeposition of AuNPs. Method 3 involved the drop coating of GAs-AuNPs using hydrothermal synthesis. As shown in Figure S2A, the CV curves of a bare electrode (curve a), the modified electrode with method 1 (curve b), the modified electrode with method 2 (curve c), and the modified electrode with method 3 (curve d) were obtained. The results showed that the response current of $[Fe(CN)_6]^{3-/4-}$ gradually increased in the order of curve a, curve b, curve c, and curve d, indicating that the hydrothermal synthesis of GAs-AuNPs composite is optimal, which accelerates the transfer of electrons on the electrode surface. The GAs-AuNPs composite material was synthesized with a one-pot hydrothermal method, combining graphene oxide (GO) and gold nanoparticles (AuNPs). This synthesis technique facilitates enhanced loading of AuNPs onto GAs, thereby maximizing the benefits of AuNPs in the composite.

Furthermore, the inclusion of AuNPs grants the material the capability to form covalent bonds with DNA through Au-S bonding[2, 3].

To verify the CV test results, EIS characterization was performed, as shown in Figure S2B. The R_{ct} values of the bare electrode (curve a), the electrode modified with method 1 (curve b), the electrode modified with method 2 (curve c), and the electrode modified with method 3 (curve d) were 127 Ω, 99 Ω, 37 Ω, and 17 Ω, respectively. Among them, the R_{ct} value of the electrode modified with method 3 was the smallest, indicating that the GAs-AuNPs composite greatly promotes the transfer of electrons on the electrode surface.

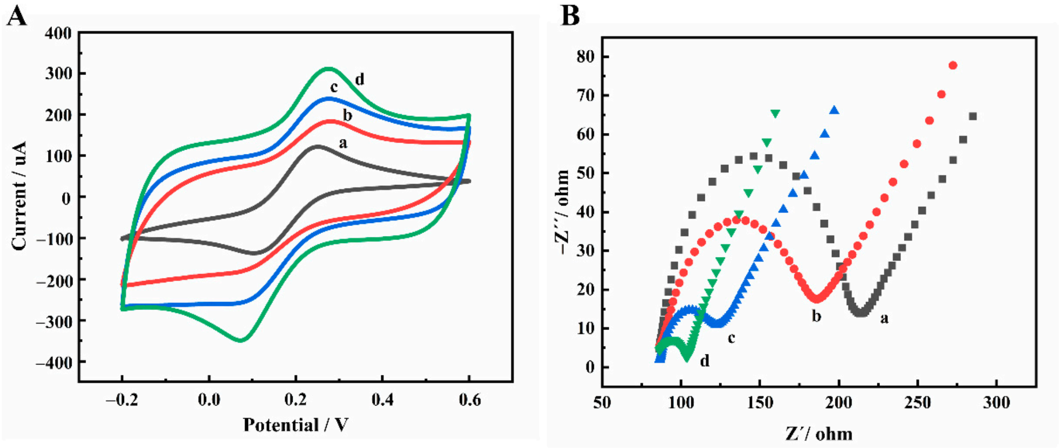

Figure S2. CVs (A) and EIS spectra (B) using different preparation methods: (a) bare electrode, (b) modified electrode with method 1, (c) modified electrode with method 2, and (d) modified electrode with method 3.

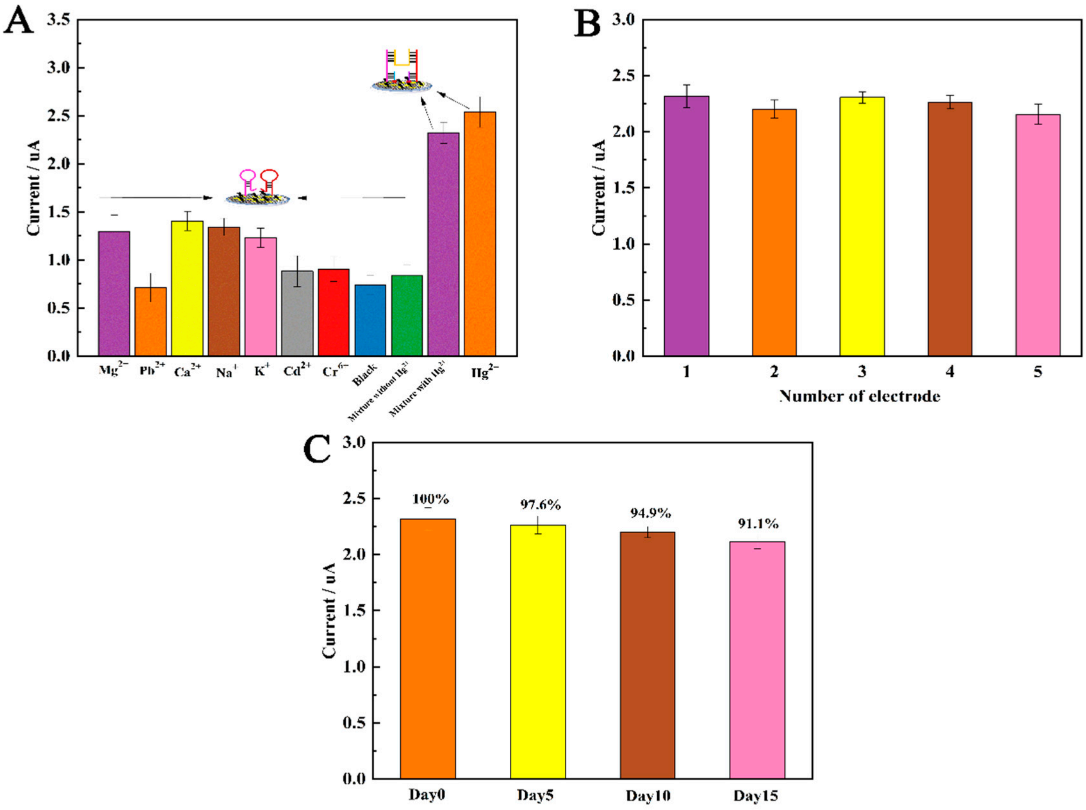

Figure S3. (A) The selectivity of the aptamer sensor to Mg^{2+}, Pb^{2+}, Ca^{2+}, Na^{+}, K^{+}, Cd^{2+}, Cr^{6+}, blank concentration, 7 kinds of interfering ions without Hg^{2+}, and 7 kinds of interfering ions with Hg^{2+} and

only Hg^{2+} (the concentration of Hg^{2+} is 1 pM, others are all 2 mM); (B) Repeatability of 5 different electrodes modified with 1 pM Hg^{2+}; (C) Stability of the aptasensor at 0, 5, 10, and 15 days.

References

1. Chen, Y.; Zhao, P.; Hu, Z.; Liang, Y.; Han, H.; Yang, M.; Luo, X.; Hou, C.; Huo, D. Amino-functionalized multilayer $Ti_3C_2T_x$ enabled electrochemical sensor for simultaneous determination of Cd^{2+} and Pb^{2+} in food samples. *Food Chem.* **2023**, *402*, 134269.
2. Karaman, C. Orange peel derived-nitrogen and sulfur Co-doped carbon dots: a nano-booster for enhancing ORR electrocatalytic performance of 3D graphene networks. *Electroanalysis* **2021**, *33*, 1356-1369.
3. Zhang, Z.; Karimi-Maleh, H. In situ synthesis of label-free electrochemical aptasensor-based sandwich-like AuNPs/PPy/$Ti_3C_2T_x$ for ultrasensitive detection of lead ions as hazardous pollutants in environmental fluids. *Chemosphere* **2023**, *324*, 138302.
